# The effect of early COVID-19 treatment with convalescent plasma on antibody responses to SARS-CoV-2

**DOI:** 10.1128/spectrum.03006-24

**Published:** 2025-06-09

**Authors:** Andy Kwan Pui Chan, San Suwanmanee, Arturo Casadevall, Shmuel Shoham, Evan M. Bloch, Kelly A. Gebo, Aaron A. R. Tobian, Andrew Pekosz, Sabra L. Klein, David Sullivan, Diane E. Griffin

**Affiliations:** 1 W. Harry Feinstone Department of Molecular Microbiology and Immunology, Johns Hopkins Bloomberg School of Public Health 25802 , Baltimore, Maryland, USA; 2 Division of Infectious Diseases, Department of Medicine, Johns Hopkins University School of Medicine 229385 , Baltimore, Maryland, USA; 3 Department of Pathology, Johns Hopkins University School of Medicine 198422, Baltimore, Maryland, USA; Universidade Federal do Rio de Janeiro, Rio de Janeiro, Brazil

**Keywords:** SARS-CoV-2, convalescent plasma, avidity, randomized controlled trial

## Abstract

At the COVID-19 pandemic onset, convalescent plasma, collected from patients recovered from severe acute respiratory syndrome coronavirus 2 (SARS-CoV-2) infection, emerged as a potential treatment option, but the effect on the patient immune response was unclear. To determine treatment effect on the infection antibody response, plasma samples were collected through 90 days from 104 SARS-CoV-2-infected participants after receiving CCP or control plasma, as part of a multicenter, double-blind, randomized, controlled early treatment trial. Enzyme immunoassay (EIA) was used to quantify binding IgG, IgA, and IgM to nucleocapsid (N) protein and IgG to the USA-WA1/2020 spike (S) protein. Avidity assessed antibody maturation. CCP transfusion boosted post-transfusion plasma levels of N-specific IgG, but not IgA or IgM and had a lesser effect on S-specific IgG. Over the first 2 weeks, both trial arm groups generated IgM, IgA, and IgG to N and IgG to S with comparable levels at 14–90 days after transfusion. Levels of IgM and IgA subsequently declined, while IgG was maintained through day 90 for both interventions. Avidity of N-specific IgG increased steadily over the 3-month follow-up in both groups, but S-specific IgG avidity improved only for those in the CCP group. Improved antibody titer correlated with improved avidity over time in both groups. Antibody levels and avidity between early ves late transfusion from symptom onset by trial arm intervention were also similar. In summary, CCP administration had no negative effects on the humoral response to SARS-CoV-2 infection and was associated with qualitatively improved antibodies with higher avidity.

**IMPORTANCE:**

Monoclonal antibody infusion prevented COVID-19 hospitalizations. However, the massive monoclonal antibody dose, near 7% of total circulating antibodies, has been shown to decrease patient generation of SARS-CoV-2 specific IgM and lower responses to vaccination, possibly by decreasing generation of high avidity neutralization epitopes near the monoclonal binding epitope. We characterized diverse antibody population quality and quantity among intervention groups in the polyclonal COVID-19 convalescent plasma (CCP) randomized control trial, effective in reducing hospitalizations by more than 50% in all participants and 80% in those receiving transfusions with symptom onset before 5 days. Importantly, we observed a greater anti-nucleocapsid compared to anti-Spike antibody level immediately after transfusion. CCP compared to controls did not alter IgG, IgA, or IgM to nucleocapsid or IgG-spike. CCP was associated with greater IgG-nucleocapsid and, to a lesser extent, IgG-spike avidity maturation over follow-up compared to control. CCP transfusions lacked negative effects on antibody levels and avidity.

## INTRODUCTION

The coronavirus 2019 (COVID-19) pandemic, caused by severe acute respiratory syndrome coronavirus 2 (SARS-CoV-2), presented unprecedented challenges to global public health. Since late 2019, various treatment strategies have been employed, from antivirals to antibody-based and immunomodulatory therapies (1). Early in the pandemic, passive transfusion of COVID convalescent plasma (CCP), defined as plasma donated from patients recovered from SARS-CoV-2 infection, was identified as a potential treatment option for individuals with COVID-19 (2, 3).

Researchers have used convalescent plasma to treat emerging virus infections because it is typically available before other potential interventions (4); however, its efficacy has been questioned. Past experience provides examples of no effect on outcome (5–8), improved outcome (9), and improved outcome with late complications (10). The latter example is particularly informative. Treatment of Junin virus-induced Argentine hemorrhagic fever with convalescent plasma decreased mortality from 16% to 1% when compared to patients who received control non-immune plasma (*P* < 0.01). However, 11% of the patients who survived after being treated with immune plasma required re-hospitalization for neurologic complications approximately 1 month later, while this was not true for survivors treated with control plasma. These data suggested that convalescent plasma caused a delay in the host antibody response to the virus and that passive antibody declined this period of low antiviral antibody allowed reactivation of virus replication in the nervous system.

Since the pioneering observation in 1964 on immune responses to phages, it has been recognized that passive IgG antibody can suppress the humoral response to antigens of the same specificity as the transferred antibody (11). The three clinically important situations for which this has been most carefully studied are maternal antibody-mediated inhibition of infant responses to vaccines, suppression of responses to RhD on erythrocytes by antibody given to Rh-negative women (12), and antibody responses to influenza virus vaccines (13, 14). For SARS-CoV-2, some clinical trials showed beneficial effects of CCP administration to COVID-19 patients as compared to control transfusion/placebo (15–21), and other trials have not (22–24). However, early in the pandemic, there was substantial variability in the quantity of antibodies present in CCP (25). The Association for the Advancement of Blood and Biotherapies clinical practice guidelines now recommends transfusing only high-titer CCP to outpatients recently infected who are at high risk of disease progression and those who are immunocompromised because these groups benefit the most (25, 26). However, because passive transfer of immune globulins during induction of an immune response can inhibit antibody responses to the targeted antigen (11, 27, 28), there is potential for both short- and long-term effects of CCP on the development of protective immunity to SARS-CoV-2.

To better understand the efficacy and effects of CCP on immune responses to SARS-CoV-2, we evaluated the longitudinal antiviral antibody responses in a single-center subgroup cohort (*n* = 104) of the total 1,181 COVID-19 outpatients who were randomized within 9 days of symptom onset to be transfused with convalescent or control plasma as part of a multicenter, double-blind, controlled trial (15). Investigation of anti-Spike-RBD-IgG at follow up visits on days 14, 28, and 90 noted no discernable total antibody level differences between control and CCP transfused participants (29). Enzyme-linked binding and avidity assays were used to measure and compare antibody quantity (IgG to N and S; IgA and IgM to N) and avidity (IgG to N and S) in plasma samples collected before transfusion, on the day after transfusion, also 14, 28, and 90 days after transfusion.

## RESULTS

### Demographic and clinical status of participants

The single center participants were outpatients with symptomatic SARS-CoV-2 infection who were randomized to receive either control (*n* = 51) or CCP (*n* = 53) between August 2020 and February 2021 (Table 1; (Fig. S1) within 9 days after onset of disease (15). Peripheral blood mononuclear cells (PBMC) and plasma were collected longitudinally from each participant at day −1 (one day prior to plasma transfusion), 0 (30 min after plasma transfusion), 14, 28, and 90 days after transfusion. Both groups had similar mean/median age, body mass index (BMI), and sex distribution. Most patients from both groups received transfusions between November 2020 and January 2021 within 6–9 days of symptom onset. The most frequent co-existing condition was hypertension with 26 (50%) in the control plasma and 17 (32%) in the CCP groups. Overall, the demographic and clinical characteristics of participants who received CCP and control plasma were similar (Table 1).

**TABLE 1:** Demographic and clinical data of trial participants in the control and CCP groups^*a*^

Characteristics	COVID-19 Convalescent plasma (*N* = 53)	Control plasma (*N* = 51)
AGE		
Median age (range) – yr	53 (33-64)	50 (32-63)
Category – no. (%)		
18-49 yr	22 (42.3%)	23 (46.1%)
50-64 yr	31 (57.7%)	28 (53.8%)
SEX		
Category – no. (%)		
F	25 (48.1%)	26 (50%)
M	28 (51.9%)	25 (50%)
BMI		
Mean BMI	29.5 (*n* = 50)	32.3 (*n* = 47)
Category – no. (%)		
≥30	18 (36%)	28 (60%)
≥35	6 (12%)	13 (8%)
EARLY/LATE TRANSFUSION		
Median time from symptom onset to transfusion – days	6	6
Category – no. (%)		
2-5 days	22 (41.5%)	22 (43.1%)
6-9 days	31 (58.4%)	29 (56.8%)
CO-EXISTING CONDITIONS		
Category – no. (%)		
None (healthy)	25 (47.2%)	17 (33.3%)
Hypertension	17 (32.7%)	26 (50%)
Asthma	3 (5.8%)	8 (15.4%)
Past Cancer	5 (9.6%)	6 (11.5%)
Tobacco use current	4 (13.5%)	1 (7.7%)
Diabetes	2 (3.8%)	8 (15.4%)
HIV	2 (3.8%)	3 (5.8%)
BMT	1 (1.9%)	0
CAD	1 (1.9%)	0
COPD	0	1 (1.9%)
Stroke	1 (1.9%)	0
VACCINATION STATUS		
Category—no. (%)		
Seronegative at screen	48 (90.5%)	42 (82.3%)
Unvaccinated (screen)	53 (100%)	45 (88.2%)
Partially vaccinated (screen)	0 (0%)	6 (11.8%)
Fully vaccinated (screen)	0 (0%)	0 (0%)
Vaccine during trial (1 dose)	9 (17.0%)	4 (7.8%)
Vaccine during trial (2 doses)	8 (15.1%)	6 (11.8%)
HOSPITALIZATION	4	2

^*a*^ Body mass index (BMI) was calculated by dividing the weight in kilograms by the square of the height in meters. BMI information was not obtained in three control and four CCP group patients. HIV, human immunodeficiency virus; BMT, bone marrow transplant; CAD, coronary artery disease; COPD, chronic obstructive pulmonary disease.

Participants vaccinated during the study (*n* = 27, 50%) included 17 (32%) in the CCP group and 10 (19%) in the control group. Six participants who received control plasma were vaccinated prior to day −1 and three participants who received CCP were vaccinated between day 14 and day 27. These nine participants were eliminated from the primary analysis. The rest of the vaccinated participants received their first dose between day 28 and day 90, thus only requiring elimination of the last study timepoint (day 90).

### Effect of plasma transfusion on the production of IgG/A/M against N protein and IgG against S protein in response to infection

**Fig 1:**
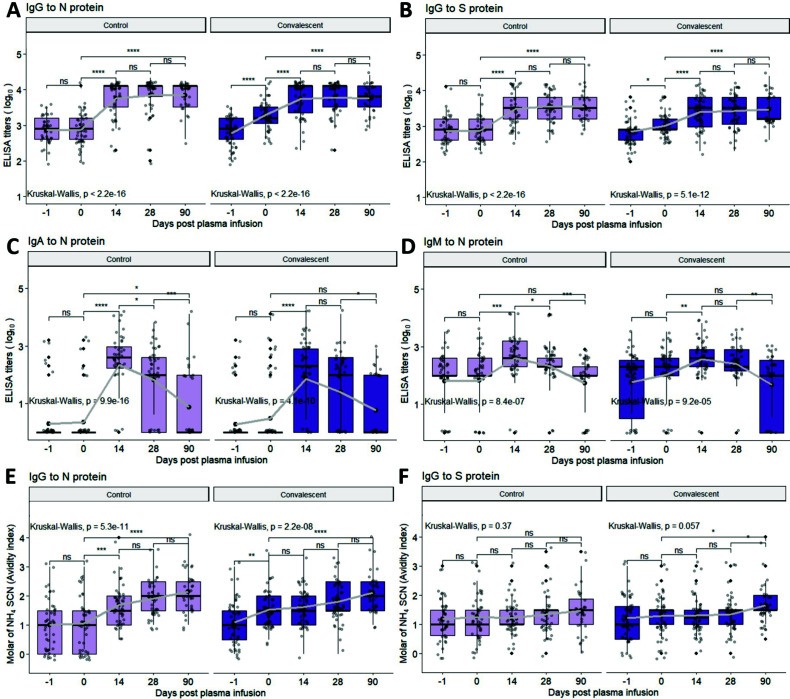
(A through D) Longitudinal analysis of antibody titers of IgG-N, IgG-S, IgA-N, and IgM-N, as well as (E and F) avidity of IgG-N and IgG for subjects that received control (light purple bars) or convalescent (dark purple bars) plasma. Statistical significance was determined by Wilcoxon test for pairwise and Kruskal–Wallis test for global comparisons: ns = non-significant, *P < 0.05, **P < 0.01, ***P < 0.001, ****P < 0.0001. Thick solid dots and lines within the quartile boxplots represent geomean and median, respectively, with gray lines in connecting the geomean EIA titers or avidity index across timepoints. Data for Fig. 1 in Table S1.

Mean EIA titers of IgG against N protein (IgG-N) and against USA-WA1/2020 S protein (IgG-S) increased from day −1 to 0 with transfusion of CCP (IgG-N: 2.77 to 3.30; IgG-S: 2.86 to 3.02) but not control plasma (Fig. 1A and B). Increases were greater for IgG-N than IgG-S and were not observed for IgA-N or IgM-N (Fig. 1C and D), indicating that CCP contained higher amounts of N-specific antiviral IgG. No significant changes in IgG-N, IgG-S, IgA-N, and IgM-N titers were observed in the control group from day −1 to 0.

To assess the effect of CCP compared to control plasma on the participant’s generated antibody response to infection, changes in mean EIA titers of IgG-N, IgG-S, IgA-N, and IgM-N between day 0 and 14 were compared. Both control and CCP groups developed similar increases in virus-specific antibodies (control: 2.85 to 3.75 in IgG-N; 2.92 to 3.49 in IgG-S; 0.36 to 2.35 in IgA-N; 1.83 to 2.60 in IgM-N; CCP: 3.29 to 3.73 in IgG-N; 3.01 to 3.40 in IgG-S; 0.49 to 1.86 in IgA-N; 2.03 to 2.56 in IgM-N) (Fig. 1C and D), with no significant differences in EIA titers at day 14 between control and CCP groups. While both groups had peak mean IgG-N, IgG-S, IgA-N, IgM-N titers at day 14, only IgG-N and IgG-S titers were maintained through day 90, while IgA-N and IgM-N titers declined (Fig. 1A through D). Also, patients in both groups had some detectable IgG-N, IgG-S, and IgM-N, but not IgA-N at the time of transfusion (day −1 and 0), suggesting that more time is required to generate IgA-N than IgM-N, IgG-N, or IgG-S. Therefore, while transfusion of CCP did not further increase the longitudinal titers of IgG-N, IgA-N, IgM-N, and IgG-S, it did not hamper the development of IgG-N, IgG-S, IgA-N, and IgM-N responses compared to the control plasma group.

### Avidity of IgG for N and S proteins

Antibody avidity represents the combined strength of multivalent antibodies to different target antigens (30), and increasing avidity indicates continual B cell maturation and germinal center selection of antibody-secreting cells (31). To assess the avidity of antiviral antibody in each sample, the molar concentration of NH_4_SCN at which > 50% of antibodies were released from the N or S-lysate-coated plates was determined.

Prior to plasma transfusion, the mean avidity index of IgG-N was 1 at day-1 in the control plasma group and 1.1 in the CCP group. Avidity remained similar at day 0 (1.05) after transfusion in the control group but increased to 1.53 in the CCP group. In the control plasma group, avidity of IgG-N increased to 1.7 at day 14 and continued to increase at day 28 (1.91) and 90 (2.14) (Fig. 1E). However, IgG-S avidity showed no significant differences across timepoints in the control group (Fig. 1F). In the CCP group, the avidity of IgG-N increased slightly by day 14 (1.62) and then to 1.81 at day 28 and 2.11 at day 90 (Fig. 1E). IgG-S avidity also showed an increase from 1.35 at day 28 to 1.65 at day 90 (Fig. 1F). Therefore, CCP contained IgG-N of greater avidity than IgG-S, yet it was associated with improved IgG-S avidity maturation over time when compared to transfusion of control plasma.

### Longitudinal changes in EIA-binding antibody and avidity index in participants receiving high delta vs low delta titer CCP

**Fig 2:**
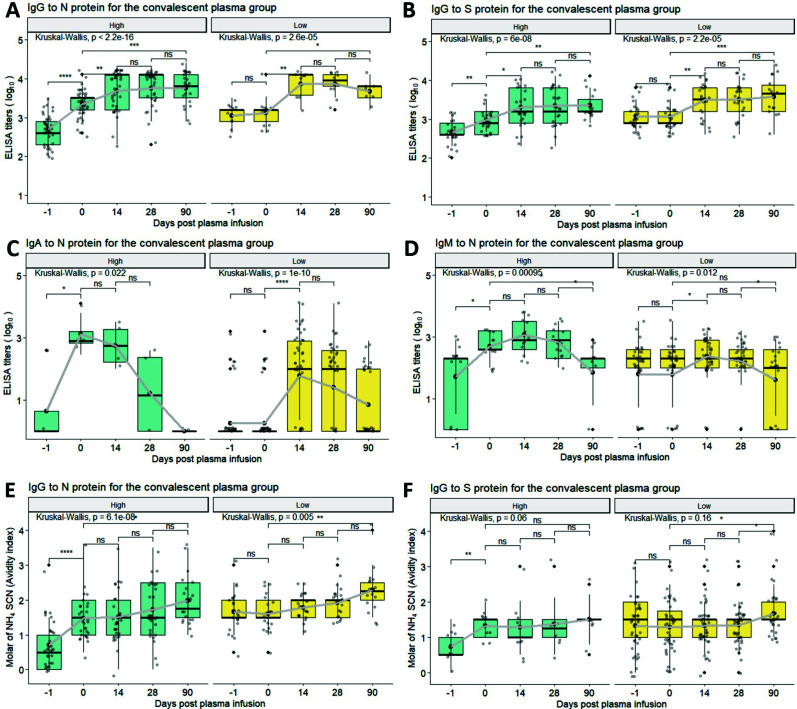
(A through D) Longitudinal analysis titers of IgG-N, IgG-S, IgA-N, IgM-N, as well as (E and F) IgG-N and IgG-S avidity, for subjects in the CCP group, which were further subdivided into high delta vs low delta titer groups based on differences between day −1 and day 0 antibody levels. Statistical significance is determined by Wilcoxon test for pairwise and Kruskal–Wallis test for global comparisons: ns = non-significant, *P < 0.05, **P < 0.01, ***P < 0.001, ****P < 0.0001. Thick solid dots and lines within the quartile boxplots represent geomean and median, respectively, with gray lines in connecting the geomean EIA titers or avidity index across timepoints. Data for Fig. 2 in Table S2.

To explore the effect of CCP quality, participants receiving CCP were subset into high delta titer and low delta titer groups (Fig. 2). High delta titer CCP referred to any IgG-N, IgG-S, IgA-N, or IgM-N titers increase from day −1 to day 0 in the analysis of longitudinal EIA titers. Similarly, avidity index changes of 0.5 or more from day −1 to day 0 served as a proxy of high delta titer CCP when evaluating the longitudinal avidity indices. As expected, the high delta group experienced a significant increase in IgG-N, IgG-S, IgA-N, and IgM-N titers (IgG-N: 2.69 to 3.34; IgG-S: 2.65 to 2.98; IgA-N: 0 to 3.20; IgM-N: 1.61 to 2.65) (Fig. 2A through D), as well as IgG-N/S avidity, from day −1 to 0 (IgG-N: 0.71 to 1.48; IgG-S: 0.72 to 1.33) (Fig. 2E and F) that was not observed in the low delta titer group. However, participants in the low delta titer group had higher baseline IgG-N/S EIA titers and avidity prior to transfusion at day −1 than the high delta titer group, suggesting seropositivity at day −1 (Fig. 2). Despite that, both groups reached similar levels of IgG-N/S titers and avidity toward day 90, except for IgG-S titers where the low delta titer group had a higher IgG-S titers than the high delta titer group at days 14, 28, and 90 (Fig. 2F).

**Fig 3:**
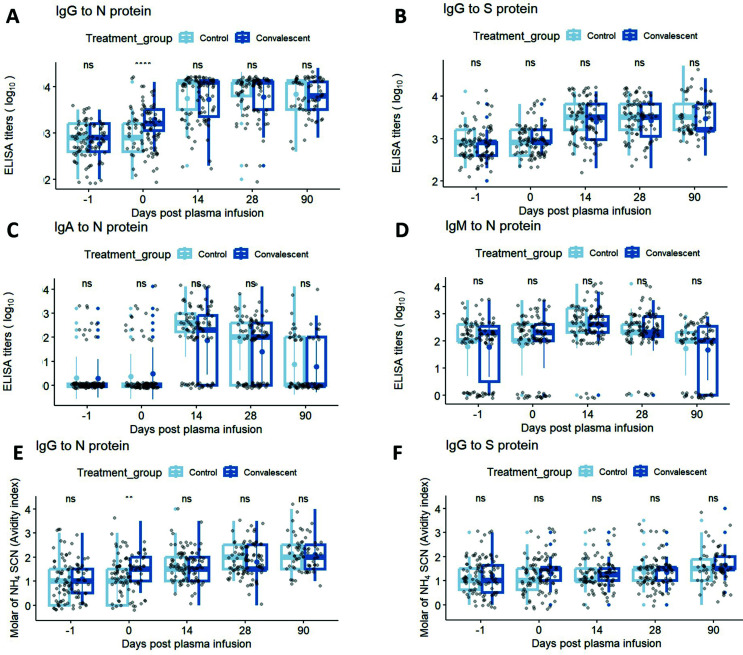
(A through D) Longitudinal analysis titers of IgG-N, IgG-S, IgA-N, IgM-N, as well as (E and F) IgG-N and IgG-S avidity, for subjects in the control vs convalescent plasma groups at each time point. Statistical significance is determined by the Wilcoxon test for pairwise comparisons: ns = non-significant, *P < 0.05, **P < 0.01, ***P < 0.001, ****P < 0.0001. Thick solid dots and lines within the quartile boxplots represent geomean and median respectively, with gray lines in connecting the geomean EIA titers or avidity index across timepoints.

In a direct comparison of control plasma to CCP recipient antibody levels and avidity by visit over time, there was a significant difference post-transfusion for IgG-N antibody levels and avidity when IgG-S antibody levels and avidity were similar post-transfusion, with possible explanation of small sample size and slightly (nonstatistical) higher pre- and post-transfusion control IgG to S protein compared to pretransfusion CCP (Fig. 3).

### Spearman analysis of the correlation between IgG-N/S and avidity

**Fig 4:**
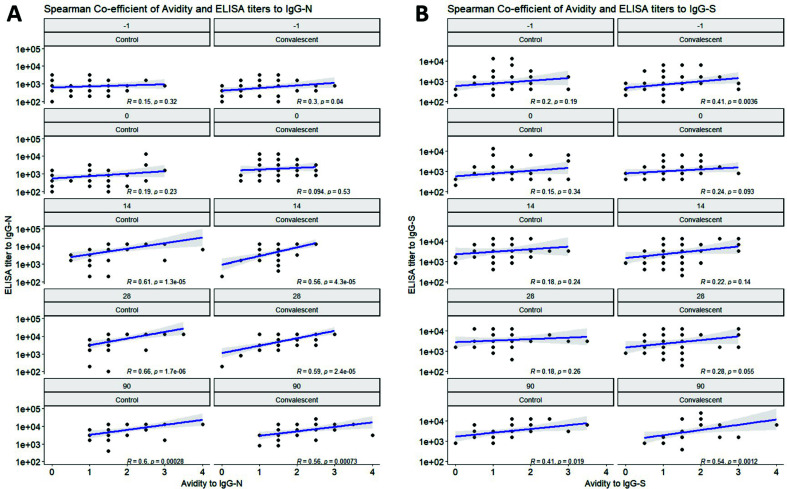
Spearman correlation of avidity index vs EIA titers to (**A**) IgG-N and (**B**) IgG-S across timepoints and between control and CCP.

Spearman co-efficient analysis of EIA titer and avidity to IgG-N/S across timepoints and treatment groups showed that IgG-N EIA and avidity were correlated at days 14, 28, and 90 in both the control plasma and CCP groups (Fig. 4A), while IgG-S was positively correlated only at day 90 (Fig. 4B), suggesting a temporal association between IgG-N/S titer and avidity, with IgG-S taking more time to be positively correlated with its titer and avidity.

### Relative avidity of IgG-N, not IgG-S, at 1M NH_4_SCN correlated with time since disease onset

**Fig 5:**
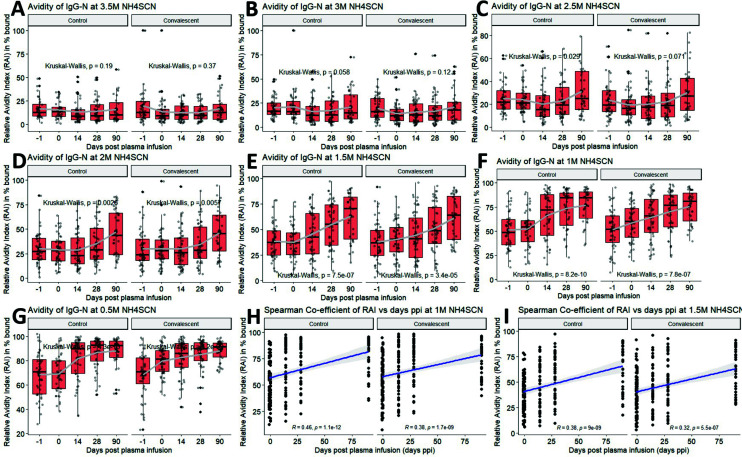
Longitudinal analysis of the relative avidity index (RAI) of SARS-CoV-2 N-specific IgG at fixed concentrations of (**A**) 3.5M, (**B**) 3M, (**C**) 2.5M, (**D**) 2M, (**E**) 1.5M, (**F**) 1M, (**G**) 0.5M NH_4_SCN, for five timepoints for control vs CCP recipients, with Spearman correlation of RAI at (**H**) 1M and (**I**) 1.5M shown. Statistical significance is determined by the Kruskal–Wallis test for global comparisons. Thick solid dots and lines within the quartile boxplots represent geomean and median respectively, with gray lines in connecting the geomean EIA titers or avidity index across timepoints.

Instead of using a range of concentrations to determine the avidity index, data can be compared using a fixed concentration of NH_4_SCN. The percentage of antibody bound upon addition of chaotropic agent vs control is plotted as the relative avidity index (RAI), where higher RAI represents better antibody avidity (30). A recent SARS-CoV-2 avidity assay using a fixed concentration of urea (4M or 7M) showed that IgG-N, not IgG-S, avidity is correlated to time since disease onset (32). We, therefore, determined the RAI of IgG-N/S with fixed concentrations of 0.5M, 1M, 1.5M, 2M, 2.5M, 3M, 3.5M NH_4_SCN, across five longitudinal sampling times for the control plasma compared to the CCP groups (Fig. 5; Fig. S2).

At high concentrations of 3.5M and 3M, mean IgG-N RAI (<20%) was very low for all samples at all times, as most N-specific antibodies were removed upon addition of a high concentration of NH_4_SCN (Fig. 5A and B). At intermediate concentrations of 2.5M and 2M, higher and lower RAI at the late and early timepoints can be differentiated (Fig. 5C and D), but it was most obvious at lower concentrations 1.5M and 1M. A linear trend of increasing RAI across time can also be detected, with RAI at 30%–50% (low avidity) at day −1 and 0 and rising to >60% RAI (Fig. 5E through G). Spearman correlation further supports that IgG-N RAI at 1M and 1.5M NH_4_SCN correlate with days since plasma transfusion in the control plasma (1M: *R* = 0.46, 1.5M: *R* = 0.38) and CCP recipients (1M: *R* = 0.38, 1.5M: *R* = 0.32) (Fig. 5H and I). Such a correlation is not found in IgG-S RAI at any fixed NH_4_SCN concentrations (Fig. S2). Overall, the data showed that the avidity of IgG-N correlates with days post plasma transfusion/time of disease onset at 1M and 1.5M NH_4_SCN. No significant differences in either antibody or avidity indices can be attributed to these confounders, except for SARS-CoV-2 vaccination status.

**Fig 6:**
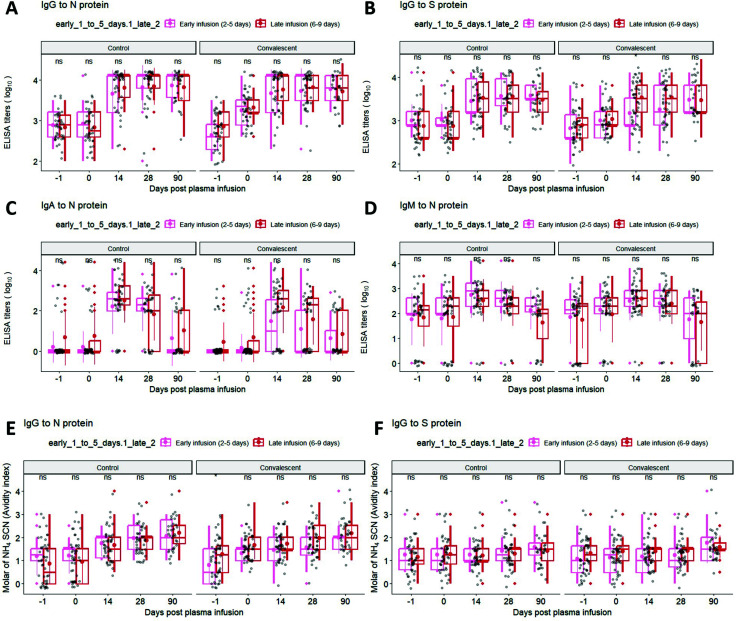
(A through D) Longitudinal analysis titers of IgG-N, IgG-S, IgA-N, IgM-N, as well as (E and F) IgG-N and IgG-S avidity, for subjects in the control vs convalescent plasma groups, which were further subdivided into early vs late infusion groups based on days post-symptom onset to days of infusion. Statistical significance is determined by the Wilcoxon test for pairwise comparisons: ns = non-significant, *P < 0.05, **P < 0.01, ***P < 0.001, ****P < 0.0001. Thick solid dots and lines within the quartile boxplots represent geomean and median respectively, with gray lines in connecting the geomean EIA titers or avidity index across timepoints. Data for Fig. 6 in Table S3.

Approximately 20 participants in each of the control and CCP groups were early (days 2–5) from symptom onset to transfusion, with approximately 30 participants in each of the late (days 6–9) from symptom onset to transfusion. Analysis of these small groups comparing antibody levels and avidity between control and CCP transfused early or late indicated no significant changes in the geometric means at day 90 (Fig. 6). While at day 14 there was a statistical difference in IgG to S protein at day 14 in the early CCP group compared to late transfusion not sustained to day 90, this may represent lower antibody levels in this subgroup compared to both early and late control plasma as well as late CCP at day 14.

## DISCUSSION

Antibody production is a critical component of the protective immune response to viral infections. Recovery from many systemic viral infections (e.g., measles, rubella, dengue) leads to induction of protective levels of antibody that are sustained life-long in the absence of re-exposure, while antibody responses to other infections (e.g., respiratory syncytial virus, rhinovirus) are short-lived (33–35). Passive transfer of immune globulins during induction of an immune response suppresses maturation of antibody-secreting cells and memory B cells with the potential for long-term effects on protective immunity and response to immunization (27, 28). Recent studies in mice have provided insight into the mechanisms of dose-dependent, epitope-specific, and FcR-independent suppression of antibody responses (27, 28). Transfusion of high doses of passive antibody decrease T follicular helper (Tfh) cell expansion, resulting in the failure of germinal center B cells to differentiate into plasma cells and memory B cells. At transfusion of lower doses of passive antibody, memory B cells are produced, but not plasma cells. Epitopes to which passive antibody is bound (usually high avidity antibodies binding to immunodominant epitopes) are unavailable for B cell receptor engagement and result in epitope masking, and these B cells do not proliferate. As such, antibodies that are subsequently produced target less dominant epitopes and are of lower avidity (28).

To determine how CCP affects the antibody response to SARS-CoV-2 infection, we analyzed the effect of CCP compared to control plasma on antibody responses of participants infected with SARS-CoV-2 enrolled in the CSSC-004 randomized controlled clinical trial (15) and found that CCP transfusion did not hinder the development of IgG-N, IgG-S, IgA-N, and IgM-N titers or IgG avidity compared to those receiving control plasma.

Transfusion of CCP rapidly increased antibody titers and avidity of IgG-N and to a lesser extent IgG-S (day −1 to day 0), but it did not lead to a further boost in longitudinal titers of IgG-N, IgA-N, IgM-N, and IgG-S when compared to control plasma at days 14, 28, and 90. These findings align with a previous study, showing that 1 h after transfusion, spike receptor-binding domain (S-RBD)-binding antibodies were significantly higher in recipients receiving CCP than control saline plus multivitamin, with this early rise in post-transfusion antibody levels being sufficient for reducing hospitalization if administered within 5 days of symptom onset. The impact of CCP on subsequent circulating antibody titers compared to control plasma groups was minimal (days 14–90) (29, 36). These findings of early effects suggest that CCP would be most useful in patients seronegative at the time of transfusion or with a compromised immune response, further supporting the use of CCP early in the course of COVID-19 infection (29).

The data show that the low delta group had higher baseline anti-N and anti-S titers, suggesting either those subjects were already mounting an antibody response before infusion or had a previous remote infection. The low delta group also mounted a blunted anti-N IgM response, which is more consistent with a secondary than primary antibody response. This group also had higher anti-S IgG titers, indicating a possible shift in the longitudinal response kinetics.

In the control group, avidity maturation for N-specific IgG is robust while that is not the case for S-specific IgG. Furthermore, consistent with other studies which showed avidity of IgG-N correlated to time of disease onset (37), avidity of IgG-N correlates with days after plasma transfusion/time of disease onset at 1M and 1.5M NH_4_SCN. This contrasts with S-specific IgG avidity that does not improve over time in the control group but does in the CCP group. This observation identifies a potential benefit from CCP transfusion beyond the immediate boost in antibody that is provided. Temporal correlations between IgG-N/S titer and avidity were also identified in both the CCP and control plasma group, with IgG-S taking longer to be positively correlated with its titer and avidity. Because IgG-S titer is positively correlated to its neutralizing potential as well as avidity (37), it indicates that patients require time to generate IgG-S of high titer, avidity, and neutralizing potential and that this may be facilitated by passive transfer of CCP early after infection. However, when comparing the responses in antibody levels or avidity between those transfused within 5 days of symptom onset to those transfused after 5 days, there were no antibody level or avidity differences in either control to CCP groups over the 3 month time period of the study.

Our results are consistent with those of another study that found administration of CCP had no deleterious effect on the adaptive immune response (38). In contrast, the administration of monoclonal antibodies (mAbs) to the S protein can have effects on the subsequent antibody response. Analysis of antibody levels in patients who received bamlanivimab, casirivimab/imdevimab, or bamlanivimab/etesevimab revealed a suppressed IgM response in those receiving mAbs relative to controls (39). Another study reported that recipients of bamlanivimab had approximately twofold lower antibody responses to vaccination (40). The differences between CCP and mAb could reflect the fact that the former involves the administration of polyclonal antibodies targeting numerous epitopes and including various isotypes, while the latter involves the administration of a large amount of a single antibody molecule binding to a single epitope. In this regard, a possible mechanism for mAb effects on the immune response is suggested by the finding that the administration of bamlanivimab skewed the repertoire of memory B cells toward non-neutralizing epitopes (41).

In conclusion, our study of participants enrolled in an outpatient clinical trial identified no negative effect of CCP on the production of antiviral antibody or antibody maturation in response to infection with SARS-CoV-2 and underscores the potential benefits of CCP, especially when given to participants who exhibited a lower COVID-specific antibody response or were seronegative at the time of transfusion. Future work could focus on whether CCP influences specific B-cell memory formation or T-cell activation; investigating cytokine profiles to assess the broader inflammatory impact of CCP.

## MATERIALS AND METHODS

### Participants

In May of 2021, 11 months into the 16 month study as part of a preplanned single center substudy characterization of COVID-19 buffy coats, 104 participants were randomly selected by an unblinded member of the data coordinating center to match intervention arm, age, and sex. No other participant characteristics were balanced. Both clinical and laboratory personnel remained blinded to outcomes until after November 2021.

### SARS-CoV-2 antigen preparation, enzyme immunoassay, and avidity measurement of antibody

The HEK293F cell line Ftet2 was transfected with plasmids containing a transposon that encodes a puromycin-resistance gene and doxycycline-inducible codon-optimized SARS-CoV-2 nucleocapsid (N) ORF (pCG144) or spike (S)** ORF (pCG146) (42). The S protein is represented by original SARS-CoV-2 S protein USA-WA1/2020, with proline substitutions that stabilize the trimeric prefusion conformation (986KV987–986PP987) and substitutions that eliminate basic amino acids at the S1/S2 cleavage site (682RRAR685–682GSAG685) (43). Cells were induced with doxycycline, lysed by three freeze-thaw cycles, placed on ice for 30 min, and centrifuged at 14,000 × *g* for 10 min before storage at −80°C.

Antibodies were quantified by EIA using clear Nunc Maxisorp 96-well plates (Thermo Fisher Scientific) coated with lysates from cells expressing S, N, or no viral protein diluted in 50 mM bicarbonate buffer pH 9.6. Coating protein concentrations and conditions were optimized for sensitivity and specificity, using pre-pandemic plasma as a negative control, and CCP as a positive control. Plates were coated overnight with N (2 µg/mL) or S (4 µg/mL) protein lysates, washed 2× with PBS containing 0.05% Tween-20 (PBST), and blocked with 5% non-fat milk in PBST overnight at 4°C. For EIA, 50 µL of plasma in 5% non-fat milk in PBST in serial dilution was incubated for 2 h. For avidity analysis, plasma at a fixed dilution of 1:50 was incubated for 2 h, washed 3×, and then incubated at room temperature for 15 min with increasing concentrations (0.5–3.5 M) of ammonium thiocyanate (NH_4_SCN) in ddH_2_O, to disrupt the antigen–antibody interactions (44). EIA or avidity plates were then washed with PBST, and horseradish peroxidase-conjugated secondary goat-antihuman IgG (Abcam; ab6858, 1:5,000), IgM (μ-chain-specific; Abcam), or IgA (α-chain-specific; Sigma-Aldrich; 1:3,000) in 5% non-fat milk in PBST was incubated for 1 h at 37°C. Plates were washed 6× with PBST and developed with 3,3′,5,5′-Tetramethylbenzidine (TMB) substrate (BD Biosciences) (50 µL/well) in the dark for 15 min before adding 2M H_2_SO_4_ (100 µL/well) as a stop solution. EIA titer is reported as the highest dilution with an optical density (OD) three times the negative control. The avidity index is reported as the concentration of NH_4_SCN required to remove more than 50% of the bound antibody. Samples with OD values < 0.3 were excluded.

### Confounders in the analysis of longitudinal antibody titers and avidity indices

Potential categorical confounders in longitudinal antibody titers and avidity indices were investigated—vaccination status, early (2–5 days) vs late (6–9 days) transfusion groups, presence of hypertension, age, BMI, and sex.

### Statistical analysis

The Wilcoxon rank-sum test or Student’s *t* tests were used to compare unpaired or paired samples between control vs CCP groups over time. Kruskal-Wallis tests were used for global comparisons. The Spearman rank correlation coefficient was used to determine correlations between samples and groups. All data graphs and statistical analysis were generated with R (version 4.1.3, 2022-03-10), using the following packages: ggplot2, tidyr, dplyr, stringr, stringi, writexl, readxl, RColorBrewer, and ggpubr.

## Data Availability

Data used to generate figures is available in the supplement materials. The authors will provide other data upon request.

## SUPPLEMENTAL MATERIAL

Supplemental material spectrum.03006-24-s0001.docx Fig. S1 and S2; Tables S1 to S3.

ASM does not own the copyrights to Supplemental Material that may be linked to, or accessed through, an article. The authors have granted ASM a non-exclusive, world-wide license to publish the Supplemental Material files. Please contact the corresponding author directly for reuse.
