# Supplementary material for: The effect of early COVID-19 treatment with convalescent plasma on antibody responses to SARS-CoV-2
Source: Microbiol Spectr. 2025 Jun 9;13(7):e03006-24. doi: 10.1128/spectrum.03006-24 (PMC12211001; doi:10.1128/spectrum.03006-24)
Supplement: Supplemental material — Fig. S1 and S2; Tables S1 to S3. [file spectrum.03006-24-s0001.docx]

**Supplementary Fig. S1** – Demographic and clinical data of peripheral mononuclear cells (PBMC) donors from the healthy control vs CCP group (n=52/group). **(A)** Age, **(B)** body mass index (BMI), **(C)** Sex, **(D)** plasma transfusion date distribution, **(E)** symptom duration (days) prior to plasma transfusion, **(F)** early vs late transfusion groups (within 2-5 and 6-9 days of symptom onset respectively), **(G)** number of vaccinated patients during study, **(H)** number of days between transfusion and first vaccination, **(I)** number of patients with co-morbidity, and **(J)** co-morbidity incidence, among the healthy control (light blue) and CCP group (dark blue), are shown. Student t-test was performed in **(A)** and **(C)** and the dotted line in **(H)** represents the end of the study at day 90.


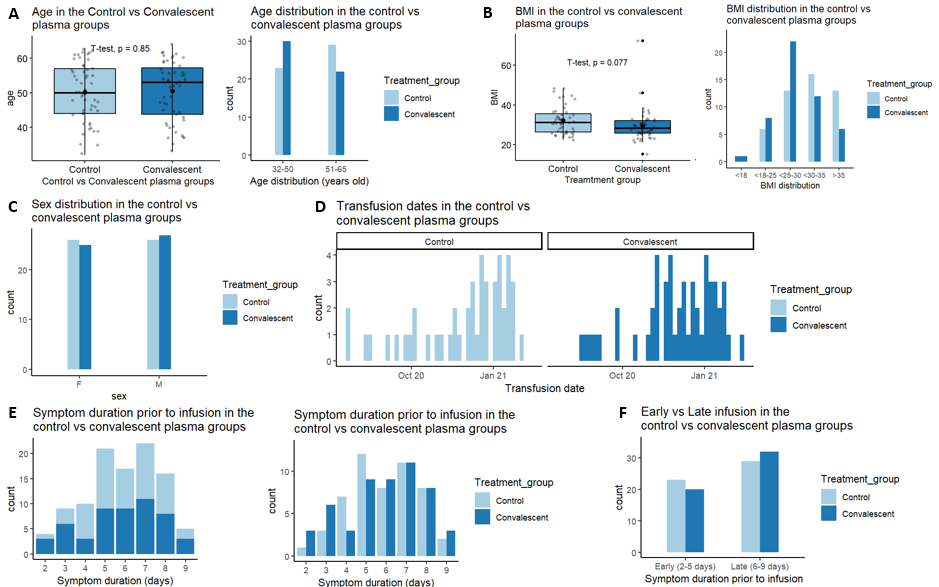


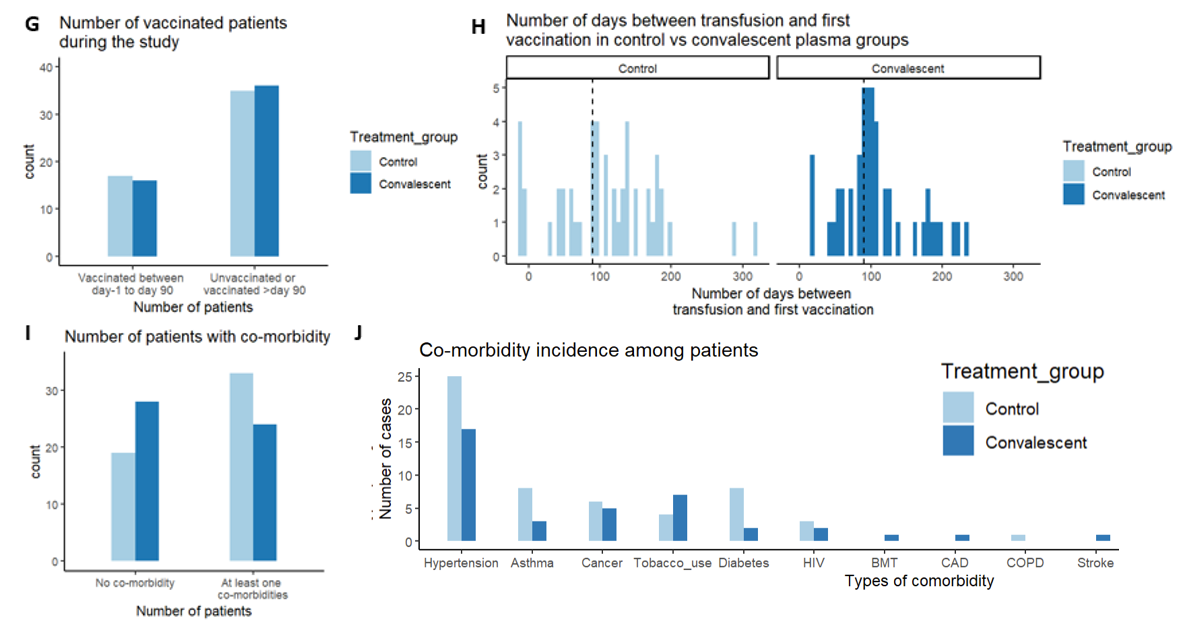


**Supplementary Fig. S2** – Longitudinal analysis of the relative avidity index (RAI) of SARS-CoV-2 S-specific IgG at fixed concentrations of **(A)** 3.5M, **(B)** 3M, **(C)** 2.5M, **(D)** 2M, **(E)** 1.5M, **(F)** 1M, **(G)** 0.5M NH_4_SCN, across five longitudinal timepoints of control vs CCP patients, with spearman correlation of RAI at **(H)** 1M and **(I)** 1.5M shown. Statistical significance is determined by Kruskal–Wallis test for global comparisons. Thick black dots and lines within the boxplots represent mean and median respectively, with grey lines in **(A-G)** connecting the mean of RAI across timepoints.


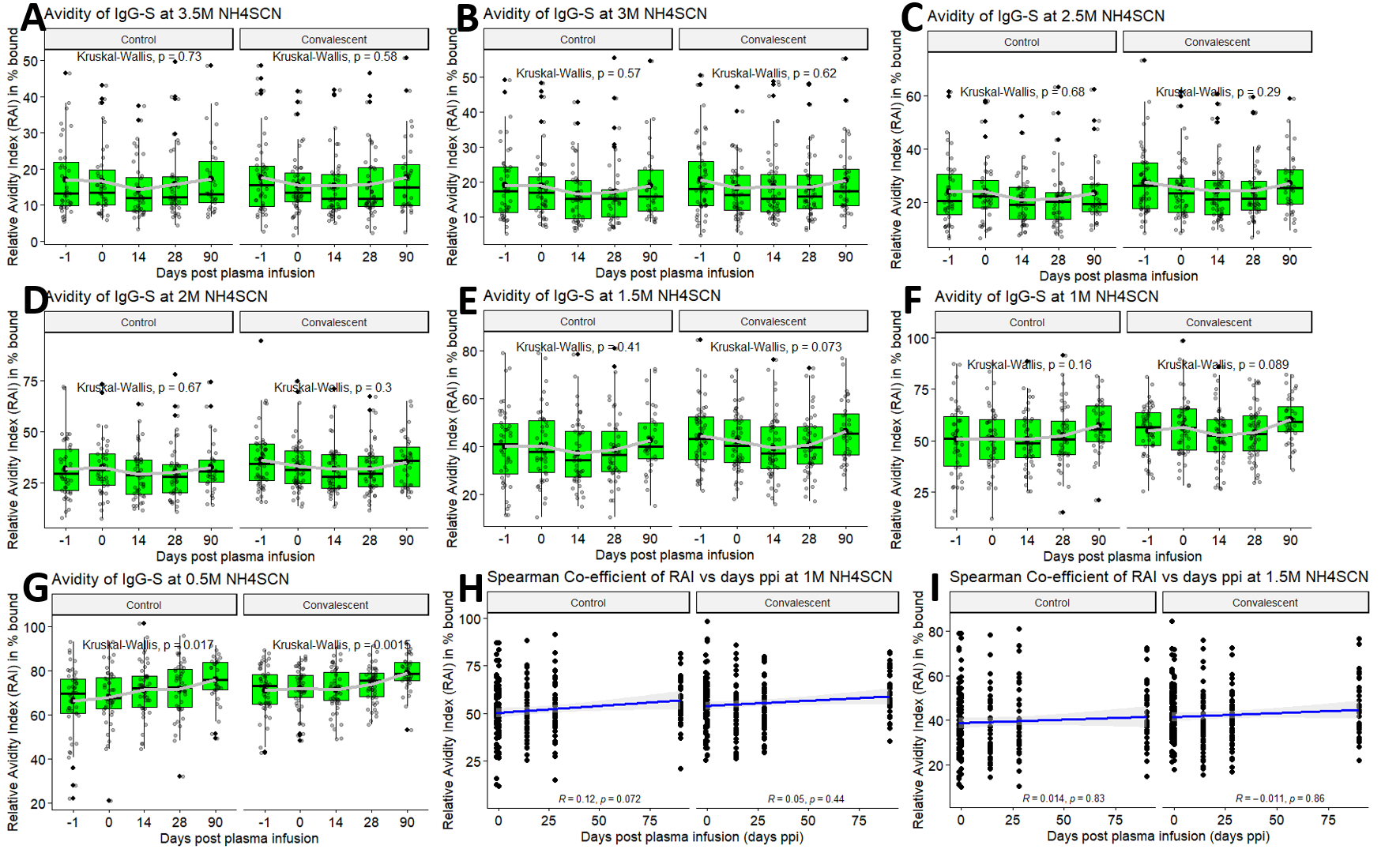


**Supplement Table 1**. Figure 1 geomean, standard deviation and median

**Supplement Table 2.** Figure 2 geomean, standard deviation and median

**Supplement Table 3.** Figure 6 geomean, standard deviation, median
